# Adult-onset central nervous system hemophagocytic lymphohistiocytosis: a case report

**DOI:** 10.1186/s12883-015-0470-6

**Published:** 2015-10-14

**Authors:** Daniel M. Pastula, Mark Burish, Gerald F. Reis, Andrew Bollen, Soonmee Cha, Jeffrey Ralph, Vanja C. Douglas

**Affiliations:** Department of Neurology, UCSF Medical Center, Box 0114, 505 Parnassus Ave, M798, San Francisco, CA 94143-0114 USA; Department of Anatomic Pathology, University of California San Francisco Medical School, San Francisco, CA USA; Department of Radiology, UCSF Medical Center, San Francisco, CA USA

**Keywords:** Hemophagocytic lymphohistiocytosis, Emperipolesis, Central nervous system

## Abstract

**Background:**

Hemophagocytic lymphohistiocytosis (HLH) is a clinical syndrome with both genetic and acquired causes characterized by elevated cytokine levels, hyperinflammation, and overactivation of lymphocytes and macrophages. It is typically a systemic disease with variable degrees of CNS involvement. Cases with predominantly central nervous system (CNS) involvement are very rare, with the vast majority of these occurring in infants and young children. This report documents a case of adult-onset CNS-HLH involving a middle-aged man.

**Case presentation:**

A 55 year-old man developed progressive left hemiparesis and aphasia over the course of several months. Brain MRI showed multifocal, mass-like enhancing lesions with increased susceptibility consistent with blood products. An extensive workup for infectious, autoimmune, and neoplastic etiologies was significant only for a markedly elevated serum ferritin at 1456 ng/mL. Two brain biopsies showed a non-specific inflammatory process. The patient was treated empirically with steroids and plasmapheresis, but he continued to suffer a progressive neurological decline and died one year after onset of neurological symptoms. Autopsy revealed profound histiocytic infiltration, perivascular lymphocytosis, and emperipolesis, compatible with CNS-HLH.

**Conclusion:**

This case report describes an exceedingly rare presentation of an adult patient with CNS predominant HLH. This diagnosis should be considered in the differential diagnosis of adults presenting with progressive brain lesions, even in the absence of typical systemic signs of HLH.

## Background

Hemophagocytic lymphohistiocytosis (HLH) is a clinical syndrome characterized by elevated cytokine levels, hyperinflammation, and overactivation of lymphocytes and macrophages [1–5]. In infants and young children, the disease is primarily caused by a genetic mutation in one of the genes responsible for cytotoxic function of natural killer cells and cytotoxic T lymphocytes. In older children, adolescents, and adults, acquired causes predominate and include infectious, neoplastic, autoinflammatory, autoimmune, and immunodeficiency etiologies. While HLH is more frequently a systemic disease with variable degrees of central nervous system (CNS) involvement, cases with predominantly CNS involvement can occur with resulting meningitis, seizures, and optic neuritis [6–12]. In such cases, the vast majority of patients are infants and young children. This report documents a case of adult-onset CNS-HLH involving a middle-aged man, which is an exceedingly rare presentation of which physicians should be aware.

## Case presentation

A 55 year-old healthy male business owner presented to a local community hospital after two months of painless left calf swelling that developed in January 2012. An ultrasound ruled out deep vein thrombosis, but an MRI suggested diffuse myositis of his left soleus muscle. Initially he was treated for cellulitis, but after failing to improve with antibiotics, the diagnosis of autoimmune myositis was entertained. A muscle biopsy showed nonspecific endomysial inflammation, and prednisone was started. His left calf swelling improved partially over the next several weeks.

In May 2012 the patient developed disequilibrium and gait unsteadiness over a period of a few days. This was followed a week later by a left foot drop, and then two weeks later by right hand parasthesias. He denied any fevers, weight loss, night sweats, or unusual rashes. He was referred to a local rheumatologist who, being concerned about the progressive symptoms, admitted him to our hospital for an expedited workup.

**Fig. 1:**
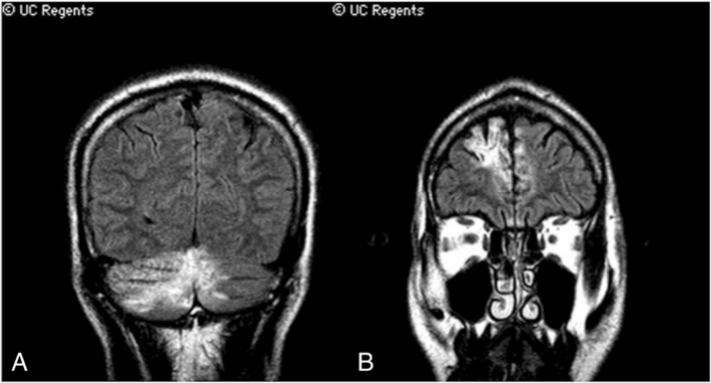
Magnetic resonance images from May 2003. Coronal fluid-attenuated inversion recovery (FLAIR) sequences through the posterior cerebellum (**a**) and the frontal lobe (**b**)

The patient was relatively healthy prior to onset. He was an avid runner and owned a successful business. Intriguingly, he had episode of right hand clumsiness in 2003 at age 46 that developed gradually and resolved slowly over months without clear etiology or any specific treatment. At the time he was found to have lesions in his right cerebellum and right frontal lobe with hemosiderin deposition deemed to be sub-acute infarcts (Fig. 1). By 2004, though, he was completely functional with no symptoms or deficits.

He had a daughter with AML and both a maternal grandmother and maternal cousin with strokes in their 40s. He was married with two children. Other than the prednisone that he had started for possible myositis, he was taking no other medications. He was not allergic to any medications.

On examination at the time of admission in June 2012, he was afebrile without rash, lymphadenopathy, or hepatosplenomegaly. He did have left lower extremity swelling. He was alert, fully oriented, with fluent speech and normal language. He had normal attention (digits forward of 6), but had poor short-term recall (0/3 at 5 min even with cues). On cranial nerve testing he was found to have a right facial droop. He had diffuse left lower extremity weakness that was worse distally in a pyramidal distribution. His deep tendon reflexes were brisk on the left compared to the right, and he had a Babinski response on the left. He had decreased sensation to pinprick over the right fifth finger. Finger-to-nose was on-target bilaterally. He had a positive Romberg test, and had a steppage gait on his left.

Labs upon admission revealed unremarkable complete blood count, chemistry panel, coagulation panel, and liver function tests. Human immunodeficiency virus (HIV) antibody was negative, rapid plasma reagin (RPR) was nonreactive, and hepatitis B and C virus serologies were negative. Erythrocyte sedimentation rate (ESR) was 6 before starting prednisone in March, and was 3 upon admission. Anti-nuclear antibody (ANA) titer was weakly positive at 1:40 and speckled, anti-double stranded DNA was negative, rheumatoid factor was negative, complement levels were normal, and both cytoplasmic and perinuclear anti-neutrophil cytoplasmic antibodies (c-ANCA and p-ANCA) were negative. Creatine kinase was elevated to 511. Ferritin was very elevated at 1456. A lumbar puncture was performed which showed 1 white blood cell; 43 red blood cells; protein of 69; glucose of 47 (serum glucose of 122); an immunoglobulin (Ig) G index of 0.6; and multiple oligoclonal bands present in both the serum and CSF (Table 1).

**Table 1:** Initial admission laboratory tests from early June 2012

Serum Test	Result
Sodium	140 mmol/L
Potassium	3.6 mmol/L
Blood urea nitrogen (BUN)	14 mg/dL
Creatinine	0.9 mg/dL
Glucose (non-fasting)	122 mg/dL
White blood cell count	3.7 X 10^9/L
Hemoglobin	13.9 g/dL
Hematocrit	40.9 %
Platelet count	204 X 10^9/L
Mean corpuscular volume	88 fL
Activated partial thromboplastin time	26.4 s
Prothrombin time	13.7 s
International normalized ratio	1.0
Total bilirubin	0.7 mg/dL
Aspartate aminotransferase (AST)	52 U/L
Alanine aminotransferase (ALT)	43 U/L
Alkaline Phosphatase	75 U/L
Ferritin	1456 ng/mL
Creatine Kinase	511 U/L
HIV antibody	Negative
Rapid plasma reagin (RPR)	Nonreactive
Hepatitis B Core Antibody	Negative
Hepatitis B Surface Antigen	Negative
Hepatitis C Antibody	Negative
Erythrocyte sedimentation rate (ESR)	3 mm/hr
Anti-nuclear antibody	Positive 1:40
Anti-double stranded DNA antibody	Negative
Anti-myeloperoxidase	Negative
Anti-proteinase 3	Negative
Rheumatoid factor	Negative
Complement C3	107 mg/dL
Complement C4	20.7 mg/dL
Complement CH50	34.6 mg/dL
Angiotensin converting enzyme	73 U/L
CSF Test	Result
Red blood cells	43 X 10^6/L
White blood cells	1 X 10^6/L
Glucose	47 mg/dL
Protein	69 mg/dL
Color	Clear
Gram stain	No organisms
Bacterial culture	No growth
M. Tuberculosis Complex PCR	Negative
IgG Index	0.6
Oligoclonal bands	Multiple bands in CSF and serum

**Fig. 2:**
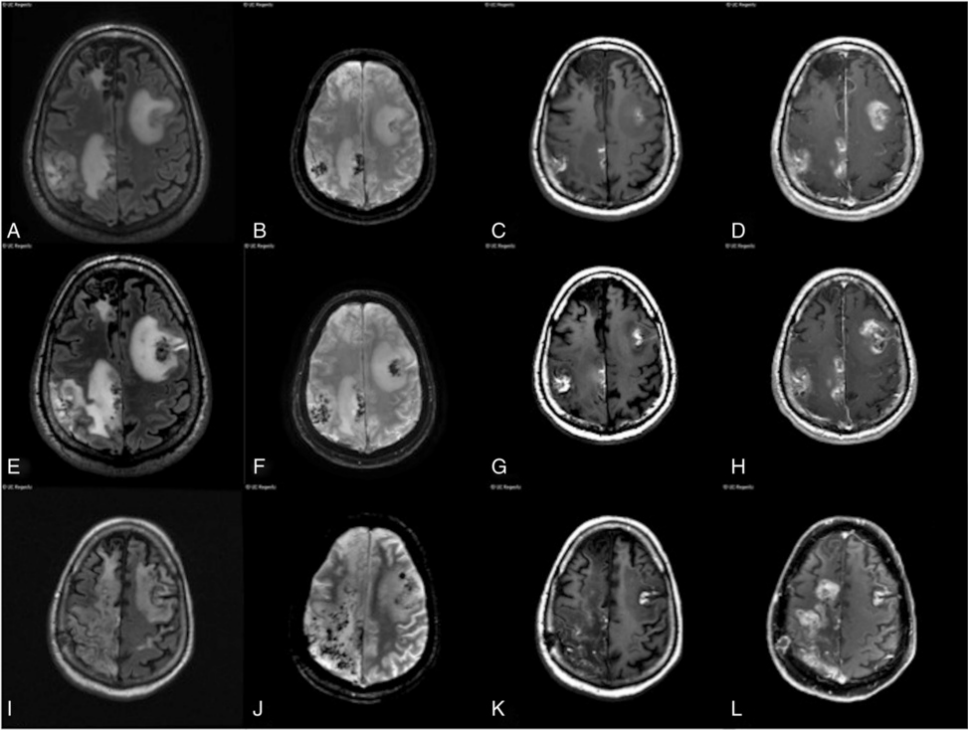
Magnetic resonance images from 2012. **a**-**d** Axial fluid-attenuated inversion recovery (FLAIR), axial 3D gradient recalled, susceptibility-weighted image, axial pre-contrast T1, and axial post-contrast T1-weighted images on June 12, 2012. **e**-**h** same sequences on June 19, 2012. **i**-**l** same sequences on August 12, 2012

Initial MRI brain showed multiple supra- and infratentorial areas of mass-like T2 hyperintensity with regional mass-effect involving cortex and juxtacortical white matter and increased susceptibility in the largest lesions consistent with blood products (Fig. 2). There was patchy, predominantly cortical enhancement within most of the lesions. The radiologic differential diagnosis at the time was sub-acute infarctions secondary to vasculopathy/vasculitis, embolic disease, or hypercoaguability; an underlying metabolic or mitochondrial disorder such as MELAS; a hemorrhagic encephalitis; or less likely a demyelinating pseudotumor.

Given these MRI findings, a broader laboratory workup was initiated to look for infectious, autoimmune, metabolic, paraneoplastic, or neoplastic processes (Table 2). This was still non-diagnostic, including normal triglycerides, fibrinogen, natural killer cell function, and soluble interleukin-2 receptor level. An MRI of the spine; MRA of the brain; CT of the chest, abdomen, and pelvis; FDG-PET whole body scan; and a transesophageal echocardiogram were also all unremarkable.

**Table 2:** Subsequent laboratory tests June-December 2013

Serum Test	Result
Lyme antibody	Negative
Triglycerides	111 mg/dL
Fibrinogen	338 mg/dL
Russel viper venom test	34.4 s
Anti beta-2 glycoprotein antibodies	Negative
Anti cardiolipin antibodies	Negative
Serum protein electrophoresis	No paraprotein spike
Vitamin B12	580 pg/mL
Lactate dehydrogenase	135 IU/L
Thyroid stimulating hormone	2.4 mIU/L
Anti thyroperoxidase antibody	Negative
Anti thyroglobluin antibody	Negative
Anti SSA antibody	Negative
Anti SSB antibody	Negative
Natural killer cell function	63 LUs
Soluble interleukin 2 receptor	713 U/mL
16 s rDNA primer set	Negative
28 s rDNA primer set	Negative
MELAS A3243G mutation: blood	Negative
Urine protein electrophoresis	Negative
Kappa/Lambda light chain ratio	0.71
CSF Test	Result
Fungal culture	No growth
Venereal disease research laboratory test	Negative
Galactomannan	Negative
Beta-D-glucan	Negative
Varicella zoster virus polymerase chain reaction	Negative
Whipple polymerase chain reaction	Negative
Cytology	Heterogeneous lymphocytes

Shortly after admission, the patient suffered a focal seizure with secondary generalization and was started on levetiracetam. He declined neurologically throughout June 2012 with worsening confusion, aphasia, and left-sided weakness. He developed occasional fevers as high as 39.9 during this month without ever having a confirmed infection.

Two brain biopsies were performed in late June 2012. The first, from a right parietal lobe lesion, showed a macrophage-rich process associated with areas of necrosis and a mild-moderate chronic inflammatory infiltrate. The second, from a right temporal lobe lesion, showed a similar macrophage-rich process involving both the cerebral cortex and white matter. No evidence of vasculitis was present in either specimen. A bone marrow biopsy was also unrevealing.

Despite this extensive workup, no clear diagnosis could be made. Suspecting an autoimmune process, IV solumedrol was administered, followed by plasmapheresis and a 5-month steroid taper. He was discharged to a skilled nursing facility in August 2012.

Despite immunosuppression, the patient continued to decline. By September 2012, he was wheelchair-bound with spastic quadriparesis, mute, and unable to maintain proper nutrition by mouth. A percutaneous endoscopic gastrostomy tube was placed in the fall of 2012. He died of aspiration pneumonia in the spring of 2013. An autopsy was performed.

### Autopsy results

**Fig. 3:**
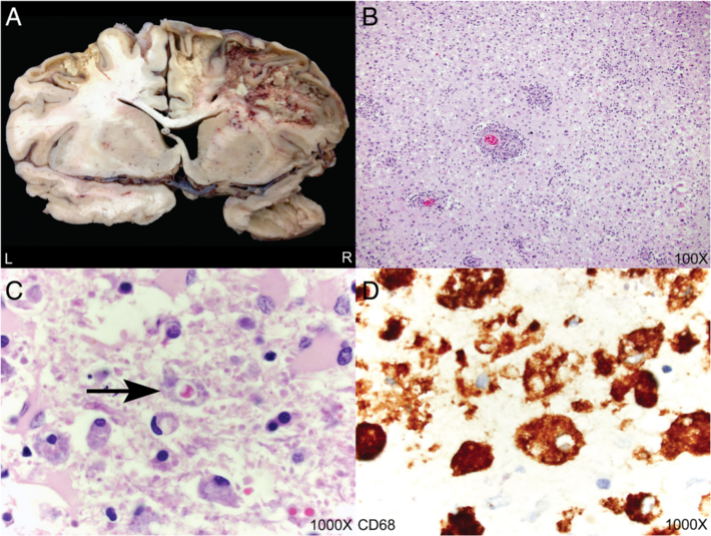
Autopsy neuropathologic findings. **a** Representative coronal section illustrating destructive lesions involving the bilateral frontal lobes and right temporal lobe. There was significant destruction of white matter parenchyma with relative preservation of the cortex. **b**-**c** Hematoxylin and eosin sections demonstrating astrogliosis and parenchymal inflammation with numerous macrophages and emperipolesis. The arrow points to a macrophage containing a red blood cell within its cytoplasm (hemophagocytosis). **d** CD68 immunohistochemistry highlighting numerous macrophages with cytoplasmic punched out spaces representing emperipolesis

Evaluation of the fresh brain showed multifocal areas of yellow discoloration and parenchymal softening at the surface, involving the bilateral frontal lobes, right parietal, right occipital, and left temporal lobes, and the right cerebellum. The fixed brain weighed 1160 g. Coronal sections of the cerebrum demonstrated multifocal parenchymal involvement with marked discoloration, cavitation, softening, and focal hemorrhage. The largest area extended from the anteriormost aspect of right frontal lobe to the posterior right occipital lobe and measured 16.5 × 8 × 4.5 cm. The disease process involved primarily the white matter and relatively spared the cortex. Similar lesions were found in the left lateral temporal lobe, right frontal gyrus, and right temporal lobe (Fig. 3a). In addition, an area of hemorrhage measuring 2.5 × 2 × 2 cm was found in the left basal ganglia, involving the putamen and white matter of the insula, and there was expansion of the left hippocampus with associated cortical thinning and tissue loss in the entorhinal cortex. A right cerebellar infarct was also identified. The spinal cord showed left lateral column atrophy spreading throughout the entire spinal column without evidence of anterior or posterior spinal root atrophy. On microscopic hematoxylin and eosin sections, the cortical sections showed profound histiocytic infiltration, perivascular lymphocytosis, and emperipolesis, compatible with CNS-HLH (Fig. 3b-c). The CD68 immunohistochemical stain demonstrated abundant macrophage infiltration and emperipolesis (Fig. 3d). Additional neuropathologic microscopic findings included a histiocytic infiltrate involving the midbrain and pons. The deltoid and quadriceps skeletal muscle showed diffuse type 2 atrophy and mild neurogenic changes, but no lymphohistiocytic infiltrate was present. Findings in other organ systems included acute and chronic aspiration pneumonia in the right lower lobe and left upper lobe of the lungs, mild atherosclerosis of the circumflex and left anterior descending coronary arteries, mild chronic cystitis with numerous friable yellow calculi, and testicular atrophy. No evidence of systemic HLH was found, including splenomegaly, hepatitis-like changes, or jaundice.

## Discussion

Hemophagocytic lymphohistiocytosis (HLH) is a hyperinflammatory syndrome characterized by overactivation of lymphocytes and macrophages in association with high levels of cytokines [1–5]. In children, the disease is relatively uncommon, with an estimated prevalence of approximately 1/100,000 [13] and incidence of 1.2/1,000,000 per year [14]. Clinically, it can be associated with fevers, hepatomegaly, splenomegaly, pancytopenia, hypertriglyceridemia, hyperferritinemia, and various neurologic manifestations. The hallmark of disease is the finding of hemophagocytosis on pathologic examination of tissues. Treatment options include immunosuppression and stem cell transplantation [1, 3–5]. The disease has primary and acquired causes. Primary HLH usually manifests as a result of defects in genes that encode proteins involved in the exocytosis of cytotoxic granules in natural killer (NK) cells, which leads to hyperactivation of the immune system. Genes encoding for perforin, syntaxin 11, Munc 13-4, and Munc 18-2 proteins have been implicated [15]. Primary HLH typically manifests in childhood. Acquired HLH is associated with various conditions, including infectious and neoplastic etiologies, which are thought to trigger the syndrome. Acquired HLH manifests predominantly in adulthood [1, 3–5]. Most cases of HLH present first with systemic involvement, [1, 3–5] though cases of central nervous system HLH (CNS-HLH) have been reported where the disease presents first with destructive lesions of the brain and spinal cord with associated meningitis, seizures, or optic neuritis [6–12]. The majority of such cases occur in the pediatric population and represent primary CNS-HLH.

In this case, the pathologic findings at autopsy are diagnostic of CNS-HLH. In retrospect, the patient’s presentation of progressive right hand clumsiness with associated brain lesions ten years earlier likely represents the earliest manifestation of this disease rather than sub-acute infarcts as it was deemed at the time. The time course and radiological similarity with subsequent lesions are also compatible with acute-on-chronic manifestations of CNS-HLH.

HLH is diagnosed clinically by either 1) having a proven genetic mutation known to be associated with HLH or 2) fulfilling 5 out of 8 clinical criteria (fever, splenomegaly, cytopenias of at least 2 cell lines, hypertriglyceridemia and/or hypofibrinogenemia, hyperferritinemia, abnormally low NK cell activity, high levels of soluble IL-2 receptor, and pathologic evidence of hemophagocytosis in tissues) [1, 3, 5, 14]. Our patient had 3 of the 8 clinical criteria: fever, hyperferritinemia, and hemophagocystosis at the time of brain biopsy. He had a transient decrease in absolute neutrophil count to 1.25 ×10^9^/L and platelets to 119 ×10^9^/L during his initial hospitalization but these values do not meet the diagnostic criteria for cytopenia in HLH. According to the HLH-94 protocol, therapy includes corticosteroids, etoposide, and cyclosporine A, with intrathecal methotrexate for those with progressive neurologic symptoms. In genetic or refractory cases, a stem cell transplant may be considered [1, 3, 5, 14, 16]. Our patient was treated empirically with corticosteroids and plasmapheresis for a presumed autoimmune condition since he did not meet clinical criteria for HLH.

The frequency of CNS involvement in HLH is unclear, especially in adults. In one small series CNS involvement was identified in up to 73 % of patients at the time of diagnosis [17]. In another, CNS involvement was present in 30 % of patients who had both HLH and an underlying autoimmune disorder [18]. In the pediatric population, neurologic symptoms are variably seen at presentation (between 13–63 % of patients), and patients may also show neuroimaging as well as CSF abnormalities [7, 9, 19–21]. Compared to patients who only have systemic symptoms, neurologic symptoms tend to occur in older patients and are associated with higher sodium levels, lower ferritin, lower levels of liver enzymes, and a worse prognosis [9, 22]. MRI findings include global volume loss, calcifications, enhancing lesions of the gray and white matter, delayed myelination, and MR spectroscopy changes (lower *N*-acetyl aspartate to creatinine ratios) [23]. CSF involvement may manifest as lymphocytic pleocytosis and elevated protein [19, 23, 24] and may indicate a worse prognosis with increased mortality [19]. Of importance to neurologists, isolated neurologic symptoms as the presenting sign of HLH have been observed in several pediatric reports [24–28] but are not particularly common [7, 9].

It is unknown whether our patient had primary or acquired HLH. While genetic causes are thought to occur mainly in children and infants, hypomorphic mutations can result in manifestation of the disease in adulthood [29]. Furthermore, defects in the gene encoding for the syntaxin-11 protein have been shown to cause both HLH and acute myeloid leukemia (AML) [30]. Intriguingly, the patient’s daughter has a history of AML, which raises consideration for a syntaxin-11 mutation and predisposition to the development of HLH. Alternatively, our patient could have been exposed to an environmental trigger (such as a virus) that could have precipitated CNS-HLH. How the therapy with solumedrol altered the inflammatory milieu prior to the autopsy is unclear; the lack of significant inflammation in the liver and other systemic organs could be due to the corticosteroid therapy. However, since the patient’s laboratories were obtained prior to initiation on solumedrol, the therapy is not a confounder in the lack of clinical/laboratory evidence necessary for a diagnosis of systemic HLH. Furthermore, the autopsy clearly demonstrates that corticosteroid therapy was not sufficient to prevent the severe destruction of brain parenchyma. Therefore, the evidence in this case is most consistent with a case of CNS-HLH presenting in adulthood.

## Conclusions

In conclusion, HLH should be considered in the differential diagnosis of adults presenting with progressive brain lesions alone or in association with recurrent fevers, splenomegaly, pancytopenia, hypertriglyceridemia, hypofibrinogenemia, and/or hyperferritinemia. Further work is needed to identify diagnostic markers of CNS-HLH and to determine the role of environmental factors in the development of HLH.

## Consent

The patient’s widow, who was his medical decision-maker in life, read this manuscript and provided consent for its publication and any accompanying images.
